# Submental Intubation in Maxillofacial Trauma Patients

**Published:** 2013

**Authors:** Amin Rahpeyma, Saeedeh khajeh Ahmadi

**Affiliations:** 1 *Oral and Maxillofacial Diseases Research Center, Faculty of Dentistry, Mashhad University of Medical Sciences, Mashhad, Iran.*; 2 *Dental Research Center ,Faculty of Dentistry, Mashhad University of Medical Sciences, Mashhad, Iran.*

**Keywords:** Intubation, Jaw fracture, Submental

## Abstract

**Introduction:**

To describe a modified technique for submental intubation in severely traumatized maxillofacial patients and to evaluate complications arising from the procedure.

**Materials and Methods:**

Submental intubation was performed in twelve patients with maxillofacial trauma ,from 2007-2012, which were operated under general anesthesia for treatment of facial fractures.

**Results:**

The patients ranged in age from 14 to 39 years. No complications due to submental intubation, such as infection, hypertrophic scarring, lingual nerve injury, hematoma, bleeding, ranula formation, or orocutaneous fistula, were observed following submental intubation.

**Conclusion:**

Submental intubation is a very useful technique in the management of maxillofacial trauma patients, with a low complication rate.

## Introduction

Traumatic events that lead to facial fractures have different etiologic factors. In many situations, treatment of facial fractures requires tracheal intubation. The fracture location, number of fracture lines, and the need for hard and soft tissue reconstructions determine the kind of intubation required. For fractures that do not involve occlusion, such as nasal, zygoma, naso-orbito-ethmoidal (NOE), frontal, and orbital blow-out fractures, oral intubation is indicated (1). For fractures that involve occlusion, such as mandibular and Lefort fractures, oral intubation inhibits appropriate resolution of the occlusion.In these situations, nasotracheal intubation is indicated (2).

However, under certain circumstances, such as persistent cerebrospinal fluid leakage, panfacial fractures, stenosis of the nasal airway by deviated nasal septum, hyperopic turbinate, and nasal polyps, nasotracheal intubation in patients with jaw fracture is not recommended (3–5).

In some cases tube exchange is possible, while tracheotomy is the preferred option in other situations. Retromolar intubation or dividing surgery into two separate procedures (nasal intubation and oral intubation) are other options (6–9). 

In the very limited cases in which the incisor teeth have been lost, an oral tube can be inserted without risk of the tube kinking during occlusion manipulation. In a few cases, extraction of a mandibular wisdom tooth will create sufficient space for the passage of the armored tube with its flexible metallic reinforcement. 

Submental intubation is a surgical method of obtaining oral intubation in cases in which the surgeon needs to evaluate occlusion during surgery (10). 

Despite the widespread use of this technique, there are few published articles on the procedure. We present herein a study of the validity of this technique in severely traumatized maxillaofacial patients.

## Materials and Methods

Under general anesthesia, after orotracheal intubation by armored tube and throat pack application, a mediolateral 2-cm length midline incision was introduced at a position 1.5–2 cm behind the mandibular lower border in the submental area. Next, dissection toward the oral cavity was performed using a thin beaked curved hemostat.

**Fig1:**
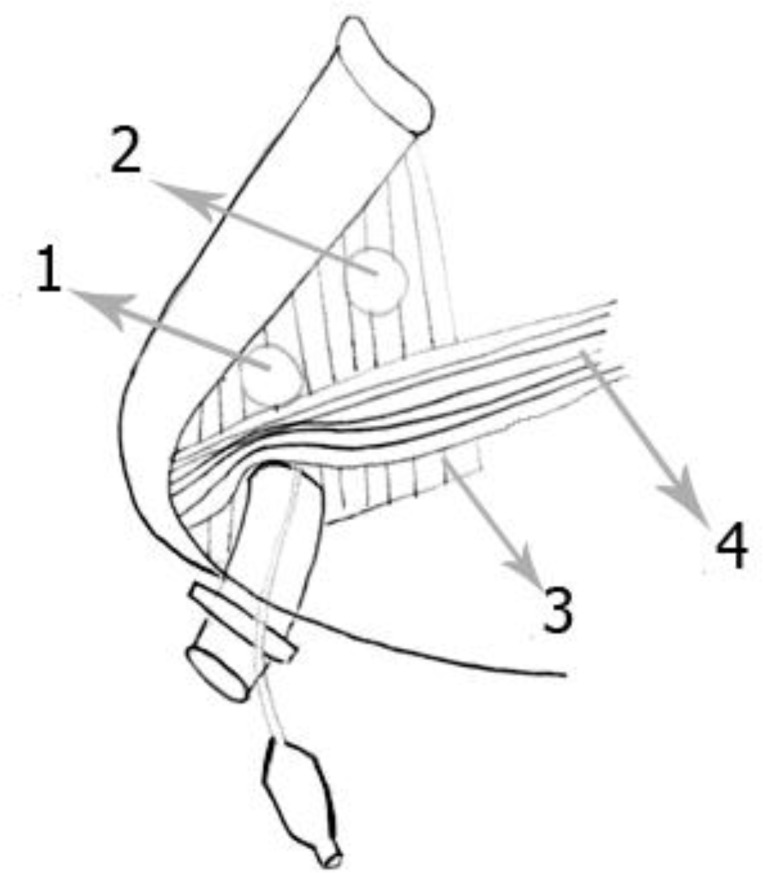
Schematic pictures of submental intubation. The tube illustrated is inserted in the midline submental region (skin is not shown). Note the myelohyoid muscle is penetrated but the anterior belly of the digaster is retracted. **1.** Submental–submandibular intubation in anterior submandibular triangle; **2.** Posterior submandibular intubation; **3.** Myelohyoid muscle; 4. Anterior belly of digaster

**Fig 2:**
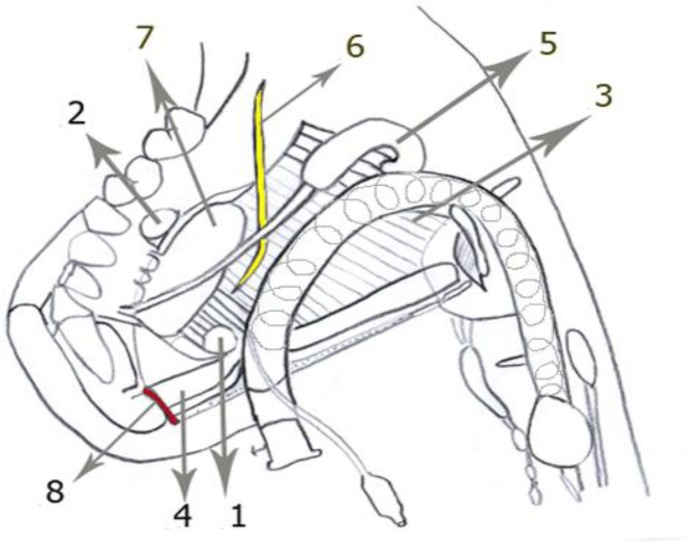
Schematic picture of submental intubation. Note the myelohyoid muscle is penetrated but the geniohyoid muscle is retracted. **1.**Submental–submandibular intubation in anterior submandibular triangle; **2.**Posterior submandibular intubation; **3.** Myelohyoid muscle; **4.** Geniohyoid muscle; **5.** Submandibular salivary gland; **6.** Lingual nerve; **7.** Sublingual salivary gland; **8.** Mandibular lingual perforation vessels

**Fig3:**
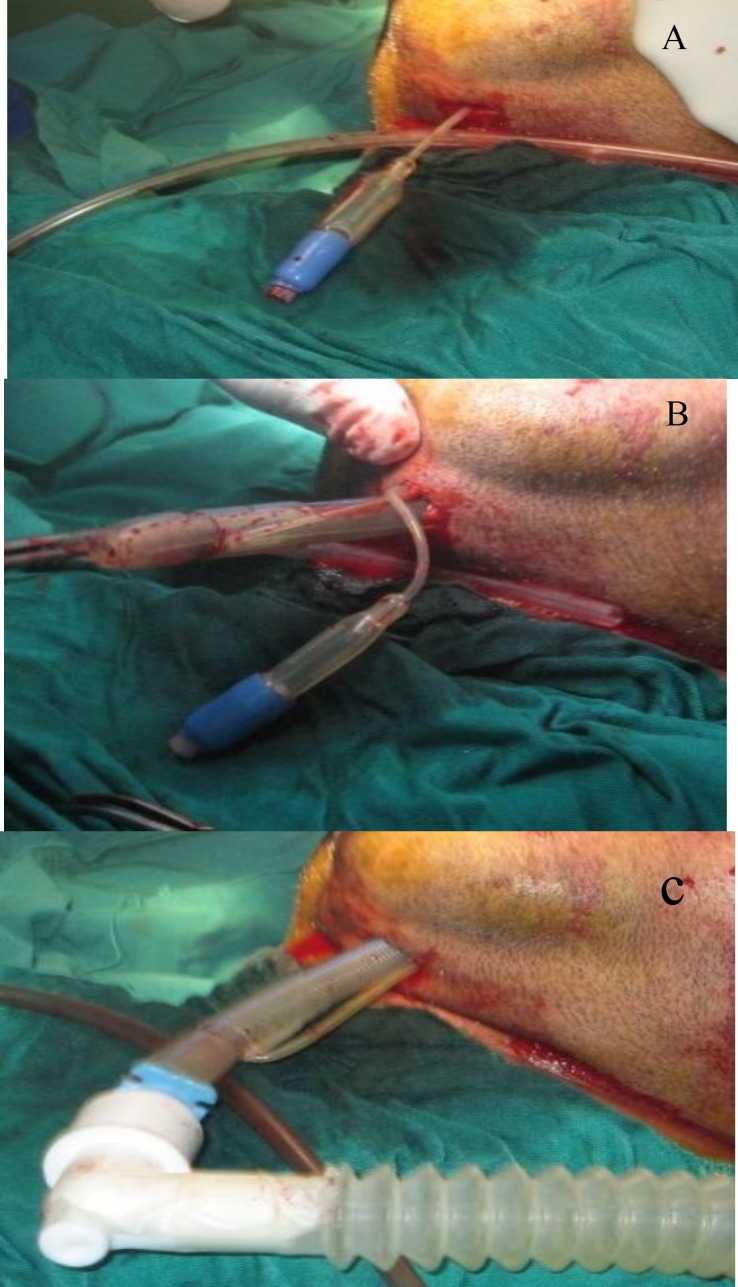
**A** and **B.** Orotracheal armored tube disconnected from ventilator, detachable connector removed, tube cuff first introduced **C.** completed submental tube

Blunt dissection was performed through the platysma, deep fascia, myelohyoid, and the floor of the mouth mucosa. The entrance point into the oral cavity was in the midline between the sublingual caruncle and the medial mandibular border. The anterior belly of the digastric and geniohyoid muscles were retracted but not penetrated (Figs.1,2). Expansion of the tissue continued until the index finger of the surgeon could pass through the tunnel from the skin to the oral cavity. The cuff of the anesthetic armored tube was first delivered through the tunnel. Then, the edge of the anesthetic tube was grasped between hemostat beaks and passed through the tunnel (Fig.3).

This motion was performed with care to prevent damage to the anesthetic tube and cuff or accidental extubation. To prevent the entry of blood or fluids into the anesthetic tube lumen, we used the cut finger of a sterile surgical glove to cover the tip, preventing fluid access to the trachea and lung tissue. The anesthetic tube was fixed by a 2-0 silk suture to the submental skin to prevent tube dislodgement. 

The introduction of the anesthetic tube from the floor of the mouth through the tunnel and outside required no more time than 1 minute, during which anesthetic connections were disconnected from the tracheal tube. Hypoxia of the patient during this short time can be avoided through prior hyperventilation by the anesthesiologist. 

At the end of the procedure, the armond tube was returned to the mouth. The submental skin was then sutured, the patient was placed at an intermaxillary fixation (IMF), and then the tracheal tube was extubated. 

The intraoral floor of the mouth exit point did not require suturing. It was possible to retain the submental tube for as long as 48 hour after the procedure (11,12).

## Results

We performed submental intubation in 12 patients with maxillofacial trauma between 2007 and 2012. The patients ranged in age from 14–39 years, and all cases had a nasal bone fracture. Fifty percent of patients also presented with mandibular fracture. Patient characteristics and fracture type are shown in (Table 1).

There were no complications due to submental intubation, such as infection, hypertrophic scarring, lingual nerve injury, hematoma, bleeding, ranula formation, or orocutaneous fistula. All patients were extubated in the operative room after fracture treatment.

**Table 1:** Age distribution and type of facial fractures in patients with submental intubation

**Case**	**Gender**	**Age**	**Type of fracture**
1	M	22	Nasal; mandibular body and angle; Lefort I fracture
2	M	27	NOE; right Zygoma; Lefort I fracture
3	F	18	Lefort III; Lefort I; mandibular symphysis and bilateral condylar fracture
4	M	14	Nasal bone and septum fracture; Lefort I; mandibular symphyseal FX
5	F	23	Left zygoma; left supraorbital rim; saddle nose; Lefort I FX
6	M	22	Lefort II; NOE FX
7	M	24	Symphysis; bilateral condyle; Lefort I; Left ZMC and nasal bone FX
8	M	35	Comminuted left malar bone FX; nasal bone mandibular left angle fracture
9	M	28	NOE; Lefort I
10	F	27	Bilateral zygoma; nasal fracture; mandibular symphyseal and angle fracture
11	M	21	Lefort I; palatal Fracture; saddle nose deformity
12	M	17	Nasal bone; right ZMC FX; Lefort I FX

M: Male, F: Female, NOE: Nasoorbitoethmoid, FX: Fracture, ZMC FX: Zygomaticomaxillary complex fracture

## Discussion

**Fig4:**
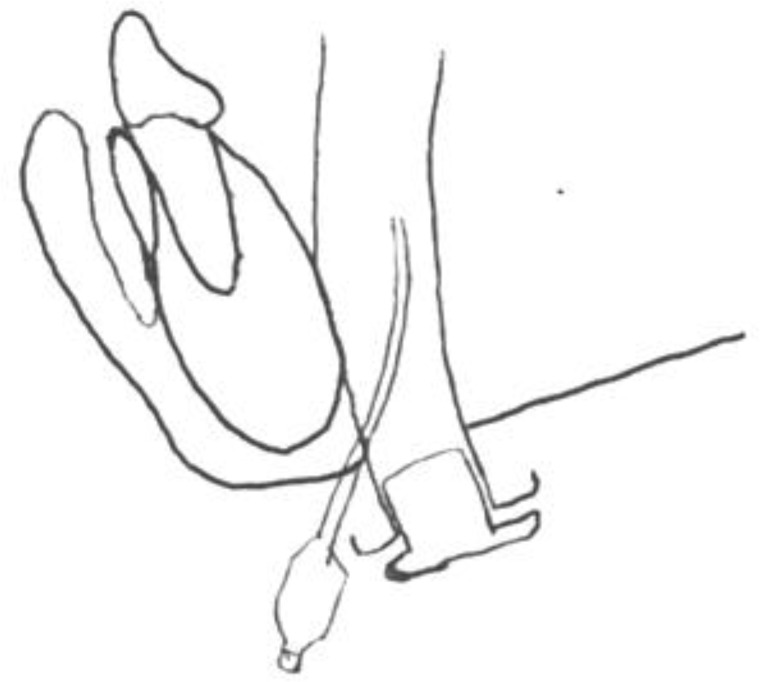
Original subperiosteal midline submental intubation described by Altemir. This system has increased risk of bleeding and hematoma due to possible mandibular lingual perforation vessel damage during subperiosteal dissection in midline, medial mandibular border

The technique of submental intubation was introduced by Altemir in 1986 (13). The first description of this technique included a midline submental skin incision and subperiosteal dissection in the mandibular bone to introduce an anesthesia tube inside the dental arch (Fig.4).

Two modifications of this technique are used more frequently today; supraperiosteal dissection and submandibular intubation (14,15). Submental intubation is very useful in particular situations, especially in panfacial maxillofacial trauma patients (16) where it may be used instead of short-term tracheotomy unless it is necessary to support the airway for prolonged periods (17).

An extraoral approach to symphysis is restricted by the presence of a submental tube. With submental intubation, occlusion can be checked during the procedure and good access can be gained to the nose through local incisions (external rhinoplasty approach) or through a bicoronal flap for more extensive reconstructions. Submental intubation can be performed using a lary- ngeal mask airway (LMA) or combitube (18,19).

Submental intubation can be used for surgical access to the pituitary gland from an oral approach and even in orthognathic surgery concomitant with rhinoplasty (20–22). The combination of submental intubation, maxillary degloving incisions, and Lefort I osteotomy provides wide access to the maxillary sinus and nasal cavity without the need for external incisions. This technique provides oral intubation without any adverse effect on occlusion because the anesthetic tube is passed lingual to the dental arch so there is no interference with occlusion.

Submental intubation avoids the risk of iatrogenic meningitis after nasotracheal intubation in patients with a recent history of cerebrospinal fluid (CSF) leakage (23). Possible complications of this technique include ranula formation, hypertrophic scarring, orocutuneous fistula, lingual nerve injury, bleeding, hematoma, and infection (24–27). 

The technique is not appropriate in patients expected to require repeated operations or those who need more than 7–14 days of postoperative ventilator support. Submental intubation can be left in place for 1–2 days and patients can then be extubated at the bedside, although longer intubations are not recommended with this technique (28). 

In our study there were no complications related to submental tube placement. The resulting scar formation was minimal and easily hidden in the submental crease. This experience is in line with comprehensive literature review in 812 patients by Jundt, on complications of submental intubation: which reported a 100% success rate with submental intubation and only minor complications (29). Tracheotomy, an alternative method, has its own complications including hemorrhage, recurrent laryngeal nerve damage, subcutaneous emphysema, tracheal stenosis, trachea-esophageal fistula, and scarring. This technique is also esthetically inferior compared with submental intubation (30).

## Conclusion

Submental intubation is a very useful technique in the management of maxillofacial trauma patients with low complications. In selected cases it may be used instead of tracheotomy, allowing possible complications of tracheotomy to be avoided.

## References

[1] Taher AA (1992). Nasotracheal intubation in patients with facial fractures. Plast Reconstr Surg.

[2] Sharma RK, Tuli P, Cyriac C, Parashar A, Makkar S (2008). Submental tracheal intubation in oromaxillofacial surgery. Indian J Plast Surg.

[3] Uma G, Viswanathan PN, Nagaraja PS (2009). Submandibular approach for tracheal intubation – a case report. Indian J Anaesth.

[4] Gibbons AJ, Hope DA, Silvester KC (2003). Oral endotracheal intubation in the management of midfacial fractures. Br J Oral Maxillofac Surg.

[5] Schütz P, Hamed HH (2008). Submental intubation versus tracheostomy in maxillofacial trauma patients. J Oral Maxillofac Surg.

[6] Martinez-Lage JL, Eslava JM, Cebrecos AI, Marcos O (1998). Retromolar intubation. J Oral Maxillofac Surg..

[7] Lima SM Jr, Asprino L, Moreira RW, de Moraes M (2011). A retrospective analysis of submental intubation in maxillofacial trauma patients. J Oral Maxillofac Surg..

[8] Lewis RJ (1992). Tracheostomies. Indications, timing, and complications. Clin Chest Med.

[9] Chandu A, Smith AC, Gebert R (2000). Submental intubation: an alternative to short-term tracheostomy. Anaesth Intensive Care.

[10] MacInnis E, Baig M (1999). A modified submental approach for oral endotracheal intubation. Int J Oral Maxillofac Surg.

[11] Green JD, Moore UJ (1996). A modification of sub-mental intubation. Br J Anaesth.

[12] MacInnis E, Baig M (1999). A modified submental approach for oral endotracheal intubation. Int J Oral Maxillofacsurg.

[13] Altemir FH (1986). The submental route for endotracheal intubation. J Maxillofac Surg.

[14] Taglialatela Scafati C, Maio G, Aliberti F, Taglialatela Scafati S, Grimaldi PL (2006). Submento-submandibular intubation: is the subperiosteal passage essential? Experience in 107 consecutive cases. Br J Oral Maxillofac Surg.

[15] Stoll P, Galli C, Wächter R, Bähr W (1994). Submandibular endotracheal intubation in panfacial fractures. J Clin Anesth.

[16] Altemir FH, Montero SH (2000). The submental route revisited using the laryngeal mask airway: a technical note. J Craniomaxillofac Surg.

[17] Caron G, Paquin R, Lessard MR, Trépanier CA, Landry PE (2000). Submental endotracheal intubation: an alternative to tracheotomy in patients with midfacial and panfacial fractures. J Trauma.

[18] Altemir FH, Montero SH, Peña M (2003). Combitube SA through submental route A technical innovation. J Craniomaxillofac Surg.

[19] Altemir FH, Montero SH (2000). The submental route revisited using the laryngeal mask airway: a technical note. J Craniomaxillofac Surg.

[20] Hejiang Z, Yunling W, Tiande Y (2011). Submental tracheal intubation for resection of recurrent giant pituitary tumor: a case report. Journal of Medical Colleges of PLA.

[21] Nyárády Z, Sári F, Olasz L, Nyárády J (2006). Submental endotracheal intubation in concurrent orthognathic surgery: a technical note. J Craniomaxillofac Surg.

[22] Chandu A, Witherow H, Stewart A (2008). Submental intubation in orthognathic surgery: initial experience. Br J Oral Maxillofac Surg.

[23] Kar C, Mukherjee S (2010). Submental intubation: an alternative and cost-effective technique for complex maxillofacial surgeries. J Maxillofac Oral Surg.

[24] Stranc MF, Skoracki R (2001). A complication of submandibular intubation in a panfacial fracture patient. J Craniomaxillofac Surg.

[25] Gordon NC, Tolstunov L (1995). Submental approach to oroendotracheal intubation in patients with midfacial fractures. Oral Surg Oral Med Oral Pathol Oral Radiol Endod.

[26] Meyer C, Valfrey J, Kjartansdottir T, Wilk A, Barrière P (2003). Indication for and technical refinements of submental intubation in oral and maxillofacial surgery. J Craniomaxillofac Surg.

[27] Stranc MF, Skoracki R (2001). A complication of submandibular intubation in a panfacial fracture patient. J Craniomaxillofac Surg.

[28] Gordon NC, Tolstunov L (1995). Submental approach to oroendotracheal intubation in patients with midfacial fractures. Oral Surg Oral Med Oral Pathol Oral Radiol Endod.

[29] Jundt JS, Cattano D, Hagberg CA, Wilson JW (2012). Submental intubation: a literature review. Int J Oral Maxillofac Surg.

[30] Garg M, Rastogi B, Jain M, Chauhan H, Bansal V (2010). Submental intubation in panfacial injuries: our experience. Dent Traumatol.

